# Effects of an eight-week pedal technique training program on functional threshold power and lower-extremity isokinetic strength in young cyclists

**DOI:** 10.3389/fspor.2026.1767183

**Published:** 2026-02-11

**Authors:** Aliye Büyükergün Kaplan, Ömercan Göksu, Milaim Berisha

**Affiliations:** 1 Faculty of Sports Sciences, Istanbul Topkapı Unıversıty, Istanbul, Türkiye; 2 Faculty of Sports Sciences, Istanbul University-Cerrahpasa, Istanbul, Türkiye; 3 Faculty of Sport Science and Movement, UBT College, Prishtine, Kosovo

**Keywords:** cycling performance, functional threshold power, isokinetic strength, pedal technique, youth cyclists

## Abstract

**Introduction:**

This study investigated the effects of an eight-week pedal technique training program on functional threshold power (FTP), knee isokinetic strength, and bilateral strength symmetry in young cyclists.

**Methods:**

Twenty-four male cyclists aged 15–17 years were assessed before and after an eight-week intervention. Anthropometric measurements, FTP, pedal analysis, and knee isokinetic strength at 60°/s, 180°/s, and 300°/s were evaluated. The training group performed pedal technique training twice per week, while the control group continued their regular training.

**Results:**

No significant changes were observed in FTP, pedal asymmetry indices, or bilateral pedal power output. The training group showed significant improvements in knee extension and flexion peak torque at 60°/s and 180°/s, whereas no meaningful changes were found at 300°/s. Isokinetic results also indicated a reduction in pre-existing strength asymmetries.

**Discussion:**

Pedal technique-focused training may improve lower-extremity isokinetic performance in young cyclists, particularly at moderate angular velocities, without significantly affecting FTP or overall pedal symmetry.

## 1 Introduction

Pedaling technique is a fundamental determinant of cycling performance and is influenced by multiple interacting factors, including cadence, exercise intensity, duration, and environmental conditions. During both training and competition, cyclists frequently experience changes in cadence and intensity, either intentionally due to tactical decisions or unintentionally in response to external factors such as braking, terrain variability, or rapid accelerations. Under these conditions, athletes are expected to maintain technical efficiency despite fluctuating mechanical demands (1). Consequently, training programs should reflect this complexity, as low-variability approaches emphasizing high intensity and low cadence may be insufficient for developing effective pedaling skills across diverse cycling conditions (1).

Previous studies have demonstrated that changes in cadence substantially affect muscle recruitment patterns and pedaling coordination, thereby influencing motor control and efficiency (1, 2). More recent evidence suggests that mechanical load may be a more decisive factor than cadence alone in determining pedal efficiency. Specifically, increasing gear ratio has been shown to improve both gross efficiency and force effectiveness, while cadence itself appears to exert a comparatively smaller influence (3). However, high-cadence cycling performed under heavy mechanical load has been reported to impair neuromuscular function, highlighting the importance of lower-extremity strength capacity in maintaining effective pedaling under demanding conditions (4).

Pedal force asymmetry represents another critical factor associated with cycling performance and injury risk. Although a certain degree of bilateral asymmetry is considered physiological, excessive differences in force production between the lower limbs have been linked to reduced performance efficiency (5) and increased injury incidence (6). Pedal force asymmetry has been shown to vary according to exercise intensity, crank torque, and bicycle setup parameters such as saddle height, suggesting that both biomechanical and neuromuscular factors contribute to asymmetrical force application (7, 8). Identifying and addressing these asymmetries through targeted training interventions may therefore help reduce mechanical inefficiencies and injury risk.

Pedal force effectiveness is commonly quantified as the ratio of effective force to total applied force throughout the crank cycle (9). During low-intensity, high-cadence cycling, the concentric–eccentric contraction cycle accelerates and pulling forces tend to diminish, whereas high-intensity, low-cadence cycling is characterized by dominant pushing forces and more stable contraction patterns (10). These varying neuromuscular demands require smooth and coordinated force application across all pedal angles. In this context, isokinetic strength becomes particularly relevant, as it supports consistent force production and facilitates efficient transitions between different cadence–intensity combinations (2).

Lower-extremity movements, including hip and knee extension and ankle dorsiflexion during the downstroke, as well as hip and knee flexion and plantar flexion during the upstroke, play a crucial role in effective pedaling mechanics (11, 12). Intra-session repeatability of lower-limb muscle activation patterns during pedaling has been demonstrated (13), supporting the reliability of assessing coordination-related outcomes. Moreover, the use of augmented feedback has been shown to modify pedaling mechanics and technique (14). Accordingly, cyclists must develop not only maximal muscular strength but also balanced inter-limb coordination and endurance in both flexor and extensor muscle groups (11).

Given the role of isokinetic strength in stabilizing transitions between varying cadences and intensities, the present study implemented a structured eight-week pedaling technique development program. To evaluate its effectiveness, key performance-related variables were assessed, including functional threshold power (FTP), bilateral knee isokinetic strength, and left–right power asymmetry. FTP testing was used both as a baseline performance indicator and to individualize training intensity, while isokinetic assessments provided insight into force production capacity under controlled angular velocity conditions. By integrating these measurements, this study aimed to determine whether targeted pedaling technique training could enhance neuromuscular adaptation and reduce force asymmetry in young cyclists.

Based on the neuromuscular demands of cycling and the role of pedaling coordination in force production, it was hypothesized that an eight-week pedal technique training program would induce velocity-specific improvements in knee extensor and flexor isokinetic strength in trained young cyclists. Specifically, greater strength adaptations were expected at moderate angular velocities (180°/s), which are more closely related to functional cycling demands. In contrast, it was hypothesized that the pedal technique–focused intervention would not result in significant changes in functional threshold power (FTP) or bilateral pedal power symmetry when compared with regular cycling training alone.

## 2 Materials and methods

### 2.1 Study design and participants

This study employed a controlled experimental design with pre- and post-test measurements (15). Twenty-four male cyclists aged 15–17 years, with a minimum cycling training experience of at least 3 years, who were actively competing at the club level and had achieved at least national-level rankings in Türkiye, voluntarily participated in the study. Participants were randomly assigned to a training group (TG, *n* = 12) or a control group (CG, *n* = 12) using a simple randomization procedure based on a computer-generated random number sequence administered by an investigator not involved in data collection or outcome assessment. All participants completed the intervention and testing procedures, and no dropouts occurred during the study period.

Inclusion criteria were as follows: male cyclists aged 15–17 years with at least 3 years of cycling training experience, active participation in club-level competitions, and achievement of at least national-level rankings in Türkiye. Exclusion criteria included any musculoskeletal injury or surgery affecting the lower extremities within the previous six months; presence of acute illness, infection, or subjective symptoms of fatigue on the testing day; use of alcohol, caffeine, or performance-enhancing substances within 48 h prior to pre- or post-testing sessions; and engagement in moderate or vigorous physical activity within 24 h before the assessments.

All procedures were conducted in accordance with the Declaration of Helsinki. Ethical approval was obtained from the Istanbul University-Cerrahpaşa Non-Interventional Clinical Research Ethics Committee (Decision No: E-74555795-05.01.04-392011; Date: 22 May 2022). Written informed consent was obtained from all participants and their legal guardians prior to participation.

Training supervision and participant adherence have now been clarified in the revised manuscript. All training sessions were supervised by the principal investigator, and adherence to the training protocol was complete, with no deviations observed.

### 2.2 Experimental procedure

The study was conducted in three phases: pre-testing, intervention, and post-testing (15). Pre-test and post-test assessments were completed within one-week periods before and after the intervention. Anthropometric measurements, functional threshold power (FTP), pedal analysis, and lower-extremity isokinetic strength assessments were performed during both testing phases.

### 2.3 Measurements

#### 2.3.1 Anthropometric measurements

Body height was measured using a stadiometer (SECA, Hamburg, Germany) with an accuracy of ±1 cm. Body mass was assessed using a calibrated electronic scale with an accuracy of ±1 CG. Body fat percentage was determined using a bioelectrical impedance analysis device, following standard manufacturer guidelines.

#### 2.3.2 Functional threshold power (FTP20) test

Functional threshold power (FTP) was assessed using the Zwift™ training software, which has demonstrated acceptable validity and reliability in previous research (16). FTP was defined as the highest mean power output that can be sustained for approximately 60 min (17). The 20-min FTP test protocol had a total duration of 45 min, including standardized warm-up and cool-down phases. During the 20 min main effort, athletes were instructed to maintain the highest sustainable power output, with a recommended cadence range of 90–95 rpm (18). FTP was calculated as 95% of the average power output recorded during the 20 min effort, and relative power output (W·kg^−^^1^) was also calculated.

All FTP assessments were performed using each participant's own bicycle mounted on an indoor trainer. All bicycles had previously undergone an individualized bike fitting process to ensure optimal and consistent cycling posture, and identical bicycle setup and fit parameters were maintained during both pre- and post-intervention assessments to minimize variability related to cyclist–bicycle interaction.

All tests were conducted during the non-competitive season. Participants refrained from intense physical activity for at least 24 h prior to testing and were instructed to maintain their habitual diet while avoiding alcohol, smoking, and nutritional supplements throughout the study period.

#### 2.3.3 Lower-extremity isokinetic strength assessment

Isokinetic strength testing of the knee extensors and flexors was performed using an isokinetic dynamometer (Cybex Humac Norm, Computer Sports Medicine Inc., Stoughton, MA, USA) following a standardized warm-up. Participants were positioned according to the manufacturer's guidelines, with the knee joint range of motion set between 0° and 90°, in accordance with established protocols (19). The device was calibrated in accordance with the manufacturer's guidelines prior to testing sessions to ensure measurement accuracy.

Concentric knee extension and flexion tests were conducted at three angular velocities:

60°·s^−^^1^ (5 repetitions × 3 sets),
180°·s^−^^1^ (10 repetitions × 3 sets), and
300°·s^−^^1^ (20 repetitions × 3 sets).

Both limbs were tested separately, with a 60 s rest interval between trials to minimize fatigue. Peak torque values were recorded in Newton-meters (Nm), and the highest value obtained from three trials at each angular velocity was used for subsequent statistical analysis. All pre- and post-intervention testing sessions were conducted at the same time of day (between 16:00 and 18:00) and under similar indoor environmental conditions to minimize the potential influence of circadian and environmental factors on neuromuscular performance.

#### 2.3.4 Pedal technique training protocol

The pedal technique training program was designed to improve force application symmetry and pedalling efficiency under varying intensity and cadence conditions (20). The protocol focused on monitoring pedalling mechanics, analyzing right–left power distribution, and enhancing neuromuscular coordination.

Each training session lasted approximately 22 min and included a standardized warm-up, structured workload intervals, active recovery periods, and a short cool-down. The protocol involved three one-minute recovery intervals between workload segments, resulting in approximately 11 min of high-intensity pedalling. Training intensity was individualized based on each athlete's FTP value determined during pre-testing.

**Figure 1:**
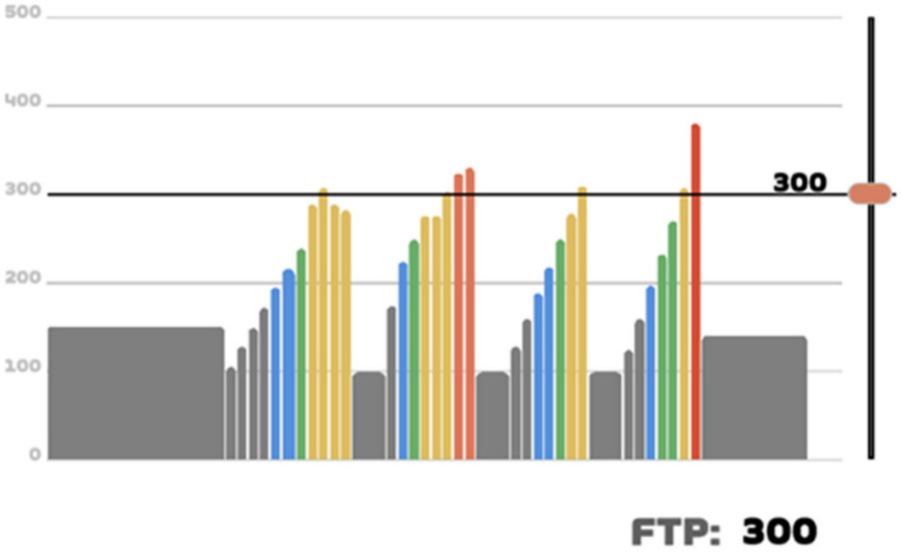
The schematic illustrates the structure of a single training session, including warm-up, workload intervals, active recovery periods, and cool-down. Training intensity was individualized according to each athlete's functional threshold power (FTP). The protocol emphasized variations in cadence and load to improve bilateral pedal force distribution and pedalling coordination.

The pedal technique training was performed twice per week for eight weeks, on days without regular team training. A schematic representation of the protocol implemented within the Zwift™ application is presented in Figure 1.

#### 2.3.5 Pedal analysis test

Pedal analysis was conducted to assess bilateral pedal force distribution and asymmetry. A standardized 3-min maximal effort test was used, and this “3-min all-out” format has been reported as a valid approach for performance-related estimation in cycling (21). Prior to testing, athletes completed a 20-min warm-up, followed by two 6 s maximal sprints separated by 5 min of rest to ensure adequate neuromuscular preparation.

During the test, athletes were instructed to maintain a cadence above 90 rpm and to cycle at maximal sustainable intensity for 3 min. Measurements were obtained using ELITE™ cycling equipment and analyzed through the My E-Training software. Average right and left pedal forces, as well as overall asymmetry indices, were recorded. Comparable 3-min device-based cycling test protocols have also demonstrated validity for physiological performance estimation (22).

### 2.4 Statistical analysis

Statistical analyses were performed using SPSS software (version 26.0; IBM Corp., Armonk, NY, USA). Descriptive statistics were calculated for all variables. Data normality was assessed using the Shapiro–Wilk test, confirming normal distribution (*p* > 0.05).

A two-way repeated-measures ANOVA was used to evaluate time (pre vs. post) × group (training vs. control) interactions. Paired-sample t-tests were applied to examine within-group changes in bilateral asymmetry measures. Effect sizes were calculated using partial eta squared (*η*²). Statistical significance was set at *p* < 0.05.

## 3 Results

### 3.1 Participant characteristics

Baseline characteristics of the training group (TG) and control group (CG) are presented in Table 1. No meaningful differences were observed between groups for age, body mass, height, or body fat percentage. Skewness and kurtosis values indicated that all variables were normally distributed (±2).

**Table 1:** Participant characteristics of the training and control groups (mean ± SD).

Variables	Training group (TG), *n* = 12	Control group (CG), *n* = 12	Total, *n* = 24
Body mass (CG)	58.5 ± 8.6	56.2 ± 8.7	57.3 ± 86
Height (cm)	170.8 ± 7.9	169.5 ± 6.7	170.1 ± 73.1
BF (%)	11.1 ± 1.2	3.2 ± 2.7	11.2 ± 20.3
Cycling experience (years)	4.0 ± 0.7	4.4 ± 1.2	4.2 ± 1.0

BF (%): body fat percentage.

### 3.2 Knee isokinetic strength–extension

Table 2 summarizes the pre- and post-intervention knee extension and flexion peak torque values at different angular velocities for the training and control groups. No statistically significant group  ×  time interaction was observed for knee extension or flexion peak torque at any angular velocity (*p* > 0.05). Effect size values (*η*²) indicated small to moderate training effects, with the largest magnitude observed for the left limb at 180°/s. For all other angular velocities and total torque values, effect sizes suggested trivial to small effects.

**Table 2:** Pre- and post-intervention knee extension and flexion peak torque values (Nm) at different angular velocities in the training and control groups.

V	Groups	N	Pre test	Post test	Changes between Pre test and Post test		
						Sig.	Eta
			Mean ± SD	Mean ± SD	*F*	*p*	*η* ^2^
60^o^/s left	CG	12	148.73 ± 41.814	149.18 ± 38.309	.233	.634	.011
	TG	12	160.67 ± 28.820	149.50 ± 34.342			
	Pre-post test [interaction time ]				.528	.475	.025
60^o^/s right	CG	12	159.45 ± 46.057	159.73 ± 34.325	.357	.557	.017
	TG	12	174.00 ± 31.482	160.58 ± 27.875			
	Pre-post test [interaction time ]				.914	.350	.042
180^o^/s left	CG	12	98.82 ± 22.982	99.09 ± 22.963	.149	.000	.969
	TG	12	97.42 ± 17.568	106.50 ± 25.400			
	Pre-post test [interaction time ]				.727	.404	.033
180^o^/s right	CG	12	106.09 ± 21.515	107.09 ± 18.716	.019	.891	.001
	TG	12	111.00 ± 22.474	100.08 ± 24.548			
	Pre-post test [interaction time ]				1.325	.263	.059
300^o^/s left	CG	12	70.91 ± 14.321	71.64 ± 14.888	.134	.718	.006
	TG	12	75.50 ± 12.362	70.50 ± 10.934			
	Pre-post test [interaction time ]				1.035	.320	.047
300^o^/s right	CG	12	78.73 ± 21.809	76.00 ± 21.424	.080	.780	.004
	TG	12	83.67 ± 21.304	67.50 ± 11.008			
	Pre-post test [interaction time ]				1.789	.195	.078
Total power left	CGr	12	811.91 ± 331.036	795.73 ± 210.205	0.15	.902	.001
	TG	12	826.83 ± 219.431	758.08 ± 209.349			
	Pre-post test [interaction time ]				.727	.404	.033
Total power right	CG	12	706.45 ± 140.870	784.91 ± 205.504	1.362	.256	.061
	TG	12	844.00 ± 260.023	815.67 ± 200.165			
	Pre-post test [interaction time]				1.245	.277	.056

Values are presented as mean ± standard deviation. TG, training group; CG, control group. F values represent the group × time interaction effects.

### 3.3 Knee isokinetic strength–flexion

Table 3 presents the pre- and post-test knee flexion peak torque values at different angular velocities for the training and control groups. A statistically significant group × time interaction was observed for knee flexion peak torque of the left limb at 180°/s (*p* < 0.05), indicating a differential response between groups over time. For all other angular velocities (60°/s and 300°/s) and for the right limb at 180°/s, no statistically significant group × time interactions were detected (*p* > 0.05).

**Table 3:** Pre- and post-test comparisons of knee flexion peak torque at different angular velocities.

D	Groups	N	Pre test	Post test	Changes between Pre test and Post test		
						Sig.	Eta
			Mean ± SD	Mean ± SD	*F*	*P*	*η* ^2^
60^o^/s left	CG	12	106.82 ± 30.626	109.91 ± 30.494	.982	.333	.045
	TG	12	96.67 ± 23.967	101.17 ± 21.908			
	Pre-post test (interaction time)				.014	.906	.001
60^o^/s right	CG	12	109.73 ± 24.540	108.64 ± 24.772	.2028	.169	.088
	TG	12	96.92 ± 20.800	109.17 ± 16.258			
	Pre-post test (interaction time)				.625	.438	.029
180^o^/sec left	CG	12	98.82 ± 22.982	99.09 ± 22.963	.14.137	.001	.402
	TG	12	69.92 ± 13.228	78.42 ± 10.783			
	Pre-post test (interaction time)				1.243	.005	.056
180^o^/s right	CG	12	78.27 ± 206.06	82.55 ± 16.403	.365	.552	.017
	TG	12	71.92 ± 14.087	81.92 ± 9.690			
	Pre-post test (interaction time)				944	.342	.043
300^o^/s left	CG	12	61.00 ± 15.388	60.27 ± 14.499	.287	.598	.013
	TG	12	53.83 ± 12.798	62.33 ± 11.531			
	Pre-post test (interaction time)				2,279	.146	.098
300^o^/s right	CG	12	60.91 ± 12.308	66.55 ± 12.801	3.685	.069	.149
	TG	12	66.55 ± 12.801	58.33 ± 6.344			
	Pre-post test (interaction time)				.062	.806	.003
Total strentgh left	CG	12	710.36 ± 278.158	696.73 ± 224.148	.000	.987	.000
	TG	12	636.25 ± 216.318	651.58 ± 193.625			
	Pre-post test (interaction time)				.084	.775	.004
Total strentgh right	CG	12	590.09 ± 150.870	685.55 ± 168.564	1.628	.216	.072
	TG	12	533.58 ± 211.690	571.58 ± 185.808			
	Pre-post test (interaction time)				.646	.431	.030

CG, control group; TG, training group.

D – variable, %. *η*^2^:effect size, X¯ ± SS: average and Standard deviation, Total Power left: total power right, Total. power. right: total power right.

Effect size analysis revealed small to moderate training effects across most conditions, with the largest effect size observed for the left limb at 300°/s. Total knee flexion strength values for both the left and right limbs did not demonstrate statistically significant group  ×   time interactions, and effect sizes suggested trivial to small training effects.

### 3.4 Functional threshold power (FTP)

Pre- and post-intervention FTP values are shown in Table 4. No significant time or group  ×   time effects were observed for relative FTP (W·CG^−^^1^) (*p* > 0.05). Effect size values indicated that the pedal technique training program had a low or negligible influence on FTP during the intervention period.

**Table 4:** Pre- and post-test comparisons of functional threshold power (FTP).

V	Groups	N	Pre test	Post test	Changes between Pre test and Post test		
						Sig.	Eta
			Mean ± SD	Mean ± SD	*F*	*P*	*η* ^2^
FTP test (watt/CG)	CG	12	3.57 ± 0.41	3.5 ± 0.24	.015	.904	.001
	TG	12	3.59 ± 0.59	3.58 ± 0.55			
	Pre-post test (interaction time)				.012	.914	.001

V, variable; % *η*^2^, partial eta square; X¯ ± SS, average and standard deviation; FTP Test, functional threshold power test.

CG, control group; TG, training group.

### 3.5 Power averages and pedal asymmetry ratios

Changes in left and right pedal power averages and pedal asymmetry ratios are summarized in Table 5. No significant differences were observed between pre- and post-tests for either group (*p* > 0.05). Group × time interaction effects were not significant for any pedal power or asymmetry variable, and corresponding effect sizes were small, indicating limited influence of the intervention on these outcomes.

**Table 5:** Pre- and post-test comparisons of pedal power averages and pedal asymmetry ratios.

V	Groups	N	Pre test	Post test	Changes between Pre test and Post test		
						Sig.	Eta
			Mean ± SD	Mean ± SD	*F*	*P*	*η* ^2^
LPPA (watt)	CG	12	149.3 ± 61.7	148.3 ± 67	.000	.994	.000
	TG	12	148.9 ± 83	148.3 ± 66.2			
	Pre-post test (interaction time)				.000	.985	.000
RPPA (watt)	CG	12	147.6 ± 70.6	143.5 ± 66.3	.001	.976	.000
	TG	12	150.2 ± 86.0	142.7 ± 60.3			
	Pre-post test (interaction time)				.035	.853	.002
PAR %	Control Group	12	51.3 ± 4.2	51.9 ± 3.8	.001	.973	.000
	TG	12	51.2 ± 4.7	52.1 ± 3.5			
	Pre-post test (interaction time)				.129	.723	.006

LPPA, left pedal power average; RPPA, right pedal power average; PAR, pedal asymmetry rate.

CG, control group; TG, training group.

### 3.6 Summary of significant changes across angular velocities

A summary of statistically significant pre–post changes across all angular velocities is presented in Table 6. Overall, significant improvements were predominantly observed at low to moderate angular velocities (60–180°/s). The training group demonstrated consistent increases in knee extension torque at these velocities, whereas flexion torque improvements at 180°/s were observed in both groups, with a more pronounced pattern in the training group.

**Table 6:** Summary of significant pre–post changes in knee isokinetic strength across angular velocities.

Angular Velocity	Movement	Group	Side(s)	Direction of change	*p*-value
60°/s	Extension	Training	R/L	↑ Increase	.011*
180°/s	Extension	Training	R/L	↑ Increase	.001*
180°/s	Flexion	Control	Right	↑ Increase	.000*
180°/s	Flexion	Control	Right	↑ Increase	.002*
180°/s	Flexion	Training	L/R	↑ Increase	.011*
300°/s	Flexion	Control	Right	↑ Increase	.034*

*Indicates a statistically significant difference (*p* < 0.05)

R, right; L, left.

No meaningful improvements were detected at 300°/s in the training group, suggesting that the pedal technique intervention preferentially influenced force production at moderate rather than high angular velocities.

## 4 Discussion

This study investigated the effects of an eight-week pedaling technique training program on functional threshold power (FTP), lower-extremity isokinetic strength, and pedal asymmetry in regularly trained young male cyclists. The main findings indicate that while the intervention did not result in significant changes in FTP or overall pedal symmetry, it contributed to improvements in inter-limb force balance, particularly under moderate angular velocity conditions during isokinetic testing.

The most notable adaptations were observed in the reduction of peak torque asymmetry at 180°/s in the training group. These findings suggest that the applied pedaling technique protocol facilitated a more balanced force production between the right and left lower extremities under conditions that closely resemble functional cycling demands. Previous research has demonstrated that bilateral asymmetry can vary considerably among cyclists, with reported values reaching up to 20%, potentially impairing mechanical efficiency and performance (23). In line with these observations, the present results support the notion that targeted technique-based interventions may help reduce excessive asymmetries, even when global performance indicators remain unchanged.

Despite these favorable neuromuscular adaptations, the intervention did not significantly improve FTP. This outcome is consistent with earlier studies indicating that short-term, technique-focused training interventions often have limited effects on high-level performance metrics such as FTP, particularly in trained populations (24, 25). FTP is a multifactorial construct influenced by cardiovascular capacity, metabolic efficiency, and peripheral muscle function (26). Consequently, improvements in pedaling coordination or force symmetry alone may be insufficient to elicit measurable changes in FTP within a relatively short intervention period.

Pedal asymmetry constituted a central outcome of the present study. Although the training group received specific feedback aimed at improving bilateral symmetry, no statistically significant reductions in pedal asymmetry indices were observed compared with the control group. This finding likely reflects the complex and multifaceted nature of pedal asymmetry, which is influenced by limb dominance, fatigue, neural activation strategies, and individual biomechanical characteristics (9, 27, 28). Such factors may limit the responsiveness of symmetry metrics to short-term interventions.

Previous investigations have highlighted the dynamic and adaptive characteristics of pedal force distribution. For example, it has been shown that directing verbal stimuli toward the dominant limb during high-intensity cycling can increase force production in the targeted limb while simultaneously reducing force output in the contralateral limb (29). These findings suggest that conscious focus on one side may inadvertently disrupt bilateral coordination. In the present study, cyclists received real-time visual and verbal feedback during pedaling tasks, encouraging them to minimize power losses across the pedaling cycle. This feedback strategy may have contributed to improved coordination and force application patterns, even in the absence of measurable changes in overall symmetry indices.

Interestingly, improvements in force distribution were more evident than changes in pedal symmetry. This observation aligns with previous reports indicating that pedal symmetry exhibits high intra- and inter-individual variability and may not consistently respond to short-term training interventions (30, 31). Moreover, some authors have questioned the relevance of pedal symmetry as a standalone performance indicator, suggesting that corrective strategies may only be necessary when asymmetry is associated with pain, injury, or functional impairment (32).

From a biomechanical standpoint, pedaling is often considered an automated and highly ingrained motor pattern, which may limit its adaptability over short time frames (24, 30). Interventions emphasizing artificial pedaling strategies, such as exaggerated circular motion or increased pull-up force, have been shown to elevate knee flexor activation without meaningful improvements in mechanical efficiency. In contrast, the present intervention focused on optimizing coordination and reducing power losses rather than enforcing a prescribed pedaling style, which may explain the selective improvements observed in isokinetic force balance.

Fatigue is another critical factor influencing force production and symmetry during cycling. Previous research has demonstrated that knee joint contributions increase substantially during high-intensity exercise performed to exhaustion (33). Additionally, experienced cyclists tend to exhibit reduced asymmetry at higher intensities, reflecting long-term neuromuscular adaptations (34). These findings support the interpretation that sustained training exposure, rather than short-term interventions, may be required to induce robust and lasting changes in pedaling symmetry and efficiency.

Recent work has also emphasized the importance of coordination between concentric and eccentric muscle actions during cycling. Improved timing between the rectus femoris, vastus medialis, and biceps femoris has been associated with enhanced joint power output under increased workloads (35). Such neuromuscular coordination may represent a more effective target for performance enhancement than symmetry-focused strategies alone. Training approaches that prioritize motor control and inter-muscular coordination may therefore provide a more stable foundation for long-term performance development.

Finally, a systematic review examining the neuromuscular effects of cadence highlighted the need for a deeper understanding of both acute and chronic adaptations to different pedaling strategies (4). The interaction between cadence, workload, and muscle activation further complicates attempts to modify symmetry through isolated mechanical or feedback-based interventions. The present findings align with this perspective, indicating that pedaling technique training may selectively influence neuromuscular characteristics without directly translating to changes in global performance outcomes.

## 5 Limitations and practical implications

### 5.1 Limitations

Several limitations of this study should be acknowledged. First, the relatively small sample size and the inclusion of only male young cyclists may limit the generalizability of the findings to other populations, such as female athletes or adult cyclists. Second, the intervention period was limited to eight weeks, which may have been insufficient to induce measurable changes in complex performance outcomes such as functional threshold power (FTP), particularly in trained athletes. Longer intervention durations may be required to observe more pronounced adaptations in aerobic performance and pedaling symmetry.

Third, although isokinetic testing provided valuable insights into angular velocity–specific strength adaptations, these laboratory-based assessments may not fully capture the dynamic and task-specific nature of force application during real-world cycling. Additionally, pedal asymmetry was assessed under controlled testing conditions, which may differ from asymmetry patterns observed during prolonged or competitive cycling efforts.

Finally, the study did not directly assess neuromuscular activation patterns, limiting the ability to identify the underlying mechanisms responsible for the observed changes in force balance and coordination. Future research incorporating neuromuscular and kinematic analyses may provide a more comprehensive understanding of the adaptations induced by pedaling technique training.

### 5.2 Practical implications

Despite these limitations, the findings offer several practical implications for coaches and practitioners working with young cyclists. The results suggest that pedaling technique training incorporating real-time feedback may contribute to improvements in lower-extremity force balance and coordination, particularly at moderate angular velocities relevant to functional cycling tasks. Such adaptations may be beneficial for reducing excessive inter-limb asymmetry, which has been associated with reduced mechanical efficiency and increased injury risk.

Importantly, the absence of significant changes in FTP indicates that pedaling technique training should not be considered a standalone method for improving aerobic performance. Instead, it may serve as a complementary strategy within a comprehensive training program that also targets cardiovascular, metabolic, and strength-related adaptations. Coaches are encouraged to integrate pedaling technique drills alongside conventional endurance and strength training, particularly during preparatory or developmental phases.

## 6 Conclusion

In conclusion, this study demonstrated that an eight-week pedal technique training program induced velocity-specific neuromuscular adaptations in trained young male cyclists. Significant improvements were observed in knee extensor and flexor strength at an angular velocity of 180°/s, whereas no meaningful changes occurred at lower (60°/s) or higher (300°/s) angular velocities. In contrast, the intervention did not result in significant changes in functional threshold power (FTP), pedal asymmetry indices, or bilateral pedal power averages when compared with the control group.

These findings indicate that pedal technique–focused training may enhance joint-level strength and neuromuscular coordination at moderate angular velocities relevant to functional cycling demands, without directly translating into improvements in global aerobic performance. Therefore, pedal technique training should be considered a complementary strategy within comprehensive training programs rather than a primary method for improving endurance performance in young cyclists.

## Data Availability

The raw data supporting the conclusions of this article will be made available by the authors, without undue reservation.
